# Ownership of Dwelling Affects the Sex Ratio at Birth in Uganda

**DOI:** 10.1371/journal.pone.0051463

**Published:** 2012-12-17

**Authors:** Bernard Wallner, Martin Fieder, Horst Seidler

**Affiliations:** 1 Department of Anthropology, University of Vienna, Vienna, Austria; 2 Department of Behavioural Biology, University of Vienna, Vienna, Austria; 3 Cognitive Science Research Platform, University of Vienna, Vienna, Austria; University of Turku, Finland

## Abstract

**Background:**

Socio-economic conditions can affect the secondary sex ratio in humans. Mothers under good environmental conditions are predicted to increase the birth rates of sons according to the Trivers-Willard hypothesis (TWH). This study analyzed the effects of ownership and non-ownership of dwellings on the sex ratio at birth (SRB) on a Ugandan sample.

**Methodology/Principal Findings:**

Our investigation included 438,640 mothers aged between 12 and 54 years. The overall average SRB was 0.5008. Mothers who live in owned dwellings gave increased births to sons (0.5019) compared to those who live in non-owned dwellings (0.458). Multivariate statistics revealed the strongest effects of dwelling ownership when controlling for demographic and social variables such as marital status, type of marriage, mothers’ age, mothers’ education, parity and others.

**Conclusions/Significance:**

The results are discussed in the framework of recent plausible models dealing with the adjustment of the sex ratio. We conclude that the aspect of dwelling status could represent an important socio-economic parameter in relation to SRB variations in humans if further studies are able to analyze it between different countries in a comparative way.

## Introduction

Usually, the sex ratio at birth (SRB = male to female live-births) exhibits a male surplus. Worldwide, the human SRB is 107 baby boys to 100 baby girls, whereas the sex ratio for the worldwide adult population changes to 101 men to 100 women [1]. A variety of biological and sociological propositions attempt to explain the imbalance at birth in terms of environmental, physiological, demographic, or socioeconomic factors – tacitly assuming that deviations from 50% need an explanation (e.g., [2], [3]). Elevated SRB values may be related, for example, to different seasonal conditions or increased temperature, to time of insemination within the menstrual cycle or copulatory frequencies, to female social dominance associated with elevated testosterone concentrations, or stressful occupational conditions [4]–[9]. Furthermore, parameters such as birth order of siblings or parity correlated with parental age have an impact on SRB rates [7], [10], [11]. Evidence is available that the number of born children is inversely related to SRB [12]. In this context, Garenne [13] analyzed more than 2 million births in the population of sub-Saharan Africa. The effect of birth order ranged significantly from 1.043 [for the first birth] to 0.993 [for the fifteenth birth] for a group of 20–39-year-old women. Increasing maternal ages, however, also negatively affected SRB rates, but only in very young [12–19 years] and old [40–49 years] mothers. Based on the influences of different marriage systems, Whiting [14] compared the SRB rates between monogamous and polygamous marriages within different Africa cultures. SRB values were lower in all polygamous systems. Studies from Ethiopia yielded conflicting results regarding the effects of the nutritional status of mothers on SRB. Gibson & Mace [15] found that their nutritional condition [measured by the arm-muscle area] was associated with increased son births. Stein et al. [16] conducted their study in other regions of Ethiopia than Gibson and Mace and used a different methodological approach. They concluded that nutritional status only marginally affected SRB rates.

The Trivers-Willard hypothesis (TWH) [17] proposes a socio-economic paradigm behind deviations in SRB rates for mammals including humans. Accordingly, mothers who are able to provide extra resources to their sons may benefit genetically by giving birth to more sons. This is because the mother’s investment is returned as a disproportionately larger number of grandchildren. How does the TWH fare when tested with real data? Overall, the results are inconclusive. One of the first studies dealing with the relation of socio-economic status and increased SRB was conducted by Norton [18], who found increased SRB values in royal families. Analogous results were confirmed in a larger sample [19] analyzing three indicators of social status – occupation, income, and education. Further interesting studies were conducted on the US population. Women who are married to men listed in the various *Who’s Who* volumes have higher SRB, but those who themselves are listed in the prominent *Social Register* have SRB rates close to 1 [20]. Similar results were obtained in a recent study among billionaires using the Forbe’s list. Male billionaires had significantly more children and more male offspring than female billionaires; moreover, women married to billionaires had significantly higher SRB rates compared to self-made female billionaires or to the whole population [21]. In present-day Venezuela, however, a weak confirmation of the TWH was found [22], while no relationship between mother’s status and elevated SRB was shown for Bari Indians [23].

The present study analyzed the influence of ownership and non-ownership of dwellings on SRB rates using a Ugandan sample of mothers. Results from former studies indicated a relationship between dwellings and reproduction. For West Germany a strong association between parenthood and home ownership was shown. However, these findings were not confirmed for the Netherlands [24]. Analyses on various housing types using longitudinal data from Finland showed that fertility was increased in single family houses [25]. As far as the authors are aware besides these results no study analyzed dwelling conditions in relation to the sex ratio at birth. The only other study considering ownership of dwelling effects (own/rented/unknown) on SRB was conducted on co-wives in Rwanda: no statistically significant relationship was found [26].

## Results

**Figure 1:**
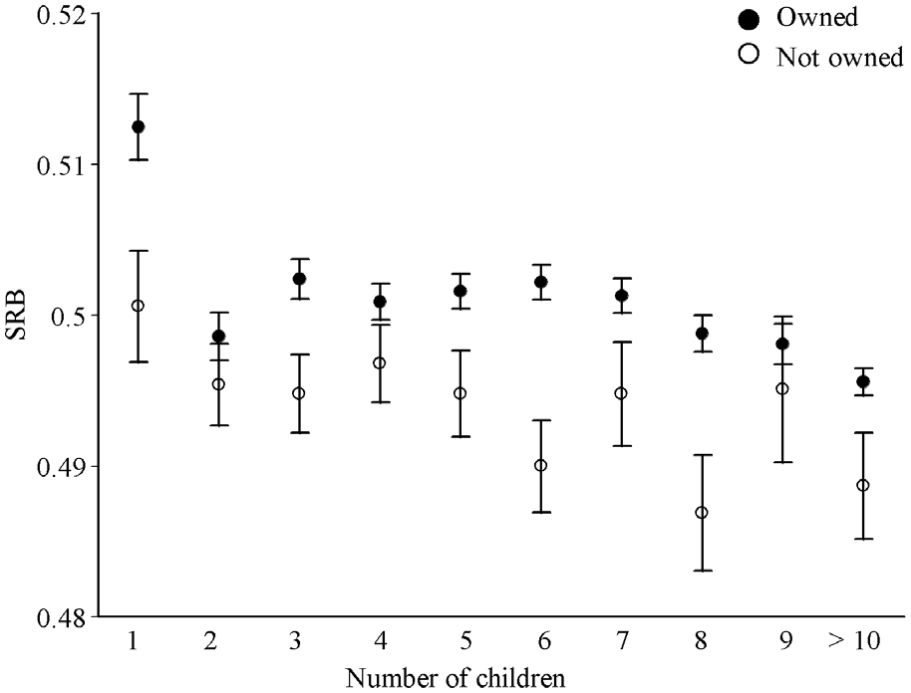
The overall SRB according to the number of children for mothers living in owned or not owned dwellings is shown. Values are mean ± SE.

The overall mean SRB of the families was 0.5008 (SE = 0.00046). For mothers who live in owned dwellings the mean SRB was 0.5019 (SE = 0.00050) and for those living in not owned dwellings it amounted to 0.4958 (SE = 0.00046); ANOVA *F* = 24.726; *P*<0.0001). SRB declined with family size, particularly from one to two children. Nonetheless, the SRB values of mothers living in owned dwellings exceeded those of mothers living in non-owned dwellings through all analyzed family sizes (Figure 1).

### SRB and Mothers Age

**Figure 2:**
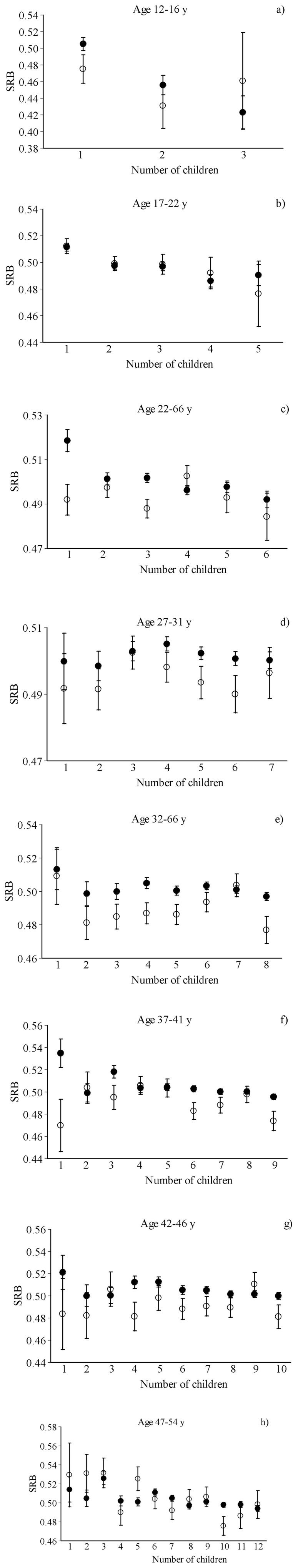
SRBs for each woman who gave birth to one, two … up to 9 children in relation to ownership of dwellings are presented. The age of these mothers is classified into eight groups (a–h). Values are mean ± SE (black symbols owned, white symbols not owned).

Analyses of SRBs classified by mothers age showed in six age-intervals increased boy births for those who resides in dwellings owned by a family member (Figure 2 a, c, d, e, f, g). Moreover, the first child born to mothers who reside in owned dwellings is more frequently a boy (found for 5 out of the 8 age intervals: Figure 2 a, c, d, f, g).

### General Linear Mixed Models (LMM) and SRB

The LMM (on basis of a normal error structure) including, type of marriage (polygamous, monogamous), educational level, ownership of dwelling, mother’s age (as fixed factors), and districts of Uganda and ethnicity (representing random factors), showed a significant positive association between SRB and monogamy respectively ownership of dwelling (Table 1).

**Table 1:** LMM of age, type of marriage, education and ownership of dwelling (ethnicity and Uganda district as random factors) regressing on SRB.

Parameter	Estimate		SE	df	T	P	95% Conf.	
							Lower limit	Upper limit
Intercept	0.5093		0.0088	122720.7955	576.002	0.0000	0.4920	0.5266
less than primary completed	−0.0136		0.0085	319982.2846	−15.977	0.1101	−0.0303	0.0031
primary completed	−0.0119		0.0085	329585.5009	−13.999	0.1616	−0.0286	0.0048
secondary completed	−0.0095		0.0090	330244.1351	−10.594	0.2894	−0.0271	0.0081
university completed ref.	0		0.0000	.	.	.	.	.
**No - monogamous**	0.0027		0.0013	142072.0762	20.374	**0.0416**	0.0001	0.0053
Yes - polygamous ref.	0		0.0000	.	.	.	.	.
**dwelling owned**	0.0051		0.0015	129721.4145	34.525	**0.0006**	0.0022	0.0081
dwelling not owned ref.	0		0.0000	.	.	.	.	.
age	0.0000		0.0001	323736.2566	−0.6004	0.5482	−0.0001	0.0001
**Parameter**			**Estimate**	**SE**				
Residual			.087360	.000214				
DistricRecode		Variance	8.705052	.000000				
Ethnicity		Variance	.000011	.000007				

Bold marked terms represent significant positive relations to SRB.

In the LMM (on basis of normal error structure) including marital status, educational level, ownership of dwelling, mother’s age (fixed factors), Uganda districts and ethnicity (as random factors), a significantly positive relation between ownership of dwelling and SRB is shown (Table 2).

**Table 2:** LMM of age, marital status, education and ownership of dwelling (ethnicity and Uganda district as random factors) is regressed on SRB.

Parameter		Estimate	SE	df	T	P	95% Conf.	
							Lower limit	Upper limit
Intercept		0.4991	0.0080	22927.3700	624.266	0.0000	0.4834	0.5147
less than primary completed		−0.0035	0.0074	64888.0075	−0.4683	0.6395	−0.0180	0.0110
primary completed		−0.0005	0.0074	93543.4871	−0.0656	0.9477	−0.0150	0.0140
secondary completed		0.0018	0.0078	250663.0698	0.2380	0.8119	−0.0134	0.0170
university completed ref.		0	0.0000	.	.	.	.	.
single or never married		−0.0031	0.0026	366712.9250	−11.984	0.2307	−0.0081	0.0019
married or living in union		0.0002	0.0020	428891.1349	0.0856	0.9317	−0.0037	0.0041
separated or divorced or spouse is absent		−0.0037	0.0025	425311.1421	−14.777	0.1395	−0.0087	0.0012
widowed ref.		0	0.0000	.	.	.	.	.
**dwelling owned**		0.0066	0.0013	2814.6815	50.361	**0.0000**	0.0040	0.0092
dwelling not owned ref.		0	0.0000	.	.	.	.	.
age		0.0000	0.0001	420327.2836	−0.2713	0.7862	−0.0001	0.0001
**Parameter**			**Estimate**	**SE**				
Residual			.093554	.000201				
Ethnicity	Variance		.000024	.000011				
DistricRecode	Variance		.000002	.000003				

Bold marked term represents a significant positive relation to SRB.

### Generalized Linear Models (GLM) and Number of Children

In the following GLM based on a quasi-poisson error structure, which included type of marriage (polygamous, monogamous), educational level, ownership of dwelling, mother’s age. All independent variables were significantly associated with number of children. Decreases in parameter estimates suggest that higher education is corroborated with lower numbers of children and women advanced in years have more children. Mothers who live in monogamous relationships do have fewer children than those under polygamous conditions. Furthermore, women living in owned dwellings have more children compared to women living in non-owned dwellings (Table 3).

**Table 3:** GLM on basis of a quasi - poisson error structure, of age, education, type of marriage and ownership of dwelling regressing on the number of children born.

				95% Conf.	
	Estimate	SE	P	Lower limit	Upper limit
(Intercept)	0.04938	0.00300	0.0001	0.04266492	0.05609114
age	0.04546	0.00008	0.0001	0.04528548	0.04563880
primary completed(ref. less than primary completed)	−0.06882	0.00186	0.0001	−0.07297171	−0.06467814
secondary completed(ref. less than primary completed)	−0.33910	0.00577	0.0001	−0.35202291	−0.32624816
university completed(ref. less than primary completed)	−0.56890	0.01637	0.0001	−0.60569963	−0.53253689
polygamous(ref. monogamous)	0.02783	0.00195	0.0001	0.02346575	0.03219536
dwelling not owned(dwelling owned ref.)	−0.18190	0.00257	0.0001	−0.18762860	−0.17613400
residual deviance: 1009518 on 673548 degrees of freedom					

All independent variables are significantly related to number of children.

A further GLM based on quasi-poisson error structure using marital status, educational level, ownership of dwelling, mother’s age, showed again significant associations between independent variables and the number of children. Again decreases in parameter estimates suggests that higher education is related to lower numbers of born children and mothers increased age is associated with more children. Parameter estimates suggest as well that married women have the highest offspring count compared to women of other marital status. Finally, women living in owned dwellings have more children compared to those who live in non-owned dwellings (Table 4).

**Table 4:** GLM on basis of a quasi - poisson error structure, of age, education, marital status and ownership of dwelling regressing on the number of children born.

				95% Conf.	
	Estimate	SE	P	Lower limit	Upper limit
(Intercept)	−1.694	0.004	0.0001	−1.70274471	−1.68529752
age	0.048	0.000	0.0001	0.04817740	0.04852880
primary completed (ref. less than primary completted)	−0.046	0.002	0.0001	−0.05033728	−0.04231958
secondary completed (ref. less than primary completted)	−0.239	0.006	0.0001	−0.25065043	−0.22722372
university completed (ref. less than primary completted)	−0.462	0.017	0.0001	−0.49626580	−0.42857514
married or living in union (ref. single or never married)	1.634	0.004	0.0001	1.62579451	1.64266601
separated or divorced or spouse is absent (ref. single or never married)	1.399	0.005	0.0001	1.38842526	1.40962376
widowed (ref. single or never married)	1.438	0.005	0.0001	1.42694235	1.44834264
dwelling not owned (dwelling owned ref.)	−0.131	0.003	0.0001	−0.13672731	−0.12607364
residual deviance: 1009518 on 673548 degrees of freedom					

All independent variables are significantly related to number of born children.

Performing generalized linear models on basis of the binomial error structure on the proportion of males and females born, we found, that not owning a dwelling is significantly negative related to the proportion of male births (Table S7 and S8). This validates the results from the LMM on SRB, previously shown.

## Discussion

Women who live in dwellings they or their family own show a higher overall birth rate than those who live in none owned dwellings. Among the analyzed socio-demographic and -economic parameters, ownership effects increased SRB rates the most. However, this influence is associated with other parameters. The small effect sizes in our study are in line with other works analyzing SRB influencing factors (e.g., [27], [28]). According to such small sizes (the odds ratios in our study are all close to 1.0; see Table S7 and S8) it can be suggested that the positive effects of ownership of dwellings on SRB ratios could represent a stochastic process of fertilization. However, the robustness of our results in all applied statistical models as well as the huge sample size do allow to conclude that ownership of dwellings effects positively the SRB ratios and these influences are strongest under married and monogamous conditions.

Our study confirms previous findings on negative effects of high parity [3], [29] and positive influences of a monogamous mating system on SRB [26]. According to parity, both groups – owner and non-owner – follow the same distribution patterns, while dwelling owners have always increased SRBs. The data point on two births differs from the others insofar as that both groups clearly give birth to more daughters. This result could reflect birth order effects, as it shown for a sub-Saharan sample [30]. In the mentioned study the SRB of firstborn children was 0.511, which corresponds to our results. Furthermore, Garenne proposed that the previous sex of children increases the probability of the same sex in the following siblings. Regarding to this information we conclude for our study that the sharp decline in SRB for both mother groups could reflect partially a stopping strategy to conceive more children. Nevertheless, the ownership group showed increased SRB for two children category. Following Garenne’s rational for unisex sibling effects, the probability increases to receive more boys for the owner group whereas the opposite is shown for non-owner mothers [26]. The positive association between maternal age and SRB rates underlines the conflicting results of earlier studies. The US population, for example, lacks a maternal age effect [31], whereas in 148 polygynous countries a positive effect was documented [32]; for sub Saharan Africa [13] and a British sample [including England and Wales; 10], negative maternal age effects were related to specific age classes of the mothers. Furthermore, our study documents that marital status can have an influence on SRB, whereas the educational level of mothers have not. This contradicts a study on US natality [33]. This mentioned discrepancy between the studies may mirror the impact of different living aspects and conditions between industrialized and developing societies.

The quality of housing conditions, e.g., overcrowding or homelessness, significantly impact human health conditions [34] and female perception. In the rural Philippines, where many mothers still live in poorly constructed dwellings (differences in construction materials in walls, doors, windows, and floors and the types of toilet facilities were analyzed), the probability of children dying before age 5 is increased [35]. Perceived dwelling conditions seem to be sex-specific: in disadvantageous housing conditions, females’ perception of safety is significantly lower than men’s, who claimed to feel safe in practically any location [36]. Therefore, we suggest that non-ownership of dwellings may enhance negative feelings in mothers. This could result in physiological stress reactions, leading to decreased SRB rates. Previous studies showed that stress-inducing circumstances, such as earthquakes, economic shortcomings or stressful job conditions, are related to elevated daughter births [9], [37], [38]. In the context of economic destitution, Catalano et al. [39] showed increased death rates for male fetuses compared to female fetuses. Physiologically, glucocorticoids secreted by stressed mothers could be responsible for these deaths because males apparently react more vulnerably to these hormones than females during developmental periods [40], which would ultimately influence secondary birth rates. Another potential explanation for the relationship between ownership of dwelling and increased SRB is provided by the maternal dominance hypothesis (see [41]). This hypothesis predicts that dominant mothers’, who have elevated testosterone concentrations around the time of fertilization, do have elevated SRB values. This has been recently confirmed for non-human primates [42]. Therefore, mothers who have access to owned dwellings – whether they own them themselves or their partners own it – could be classified as wealthy, respectively, could represent a high dominant status in their social community. Both parameters, wealth and dominance, can increase SRB. Finally, our study yields different results compared to an earlier analyzing hierarchal effects on the SRB in co-wives in Rwanda [26]. The latter study used dwelling [own/rented/unknown] among other co-variables in their multivariate statistics and found no dwelling effects on SRB. Both societies, the Rwandan and Ugandan, are primarily rural and polygamous. The countries may differ in their specific home affairs and therefore, future research on SRB differences could reveal interesting results.

The main result of the recent study indicates that the ownership or non-ownership of dwellings has a strong impact on the SRB in humans. The authors therefore conclude that the socio-economic parameter dwelling represents an additional supportive parameter of the TWH. Further detailed comparative investigations on this so far neglected parameter should provide more detailed information on how parental socio-behavior and reproductive physiology is affected in both industrial and developing countries.

## Materials and Methods

We used the 10% census sample of Uganda from 2002, provided by IPUMS international (Minnesota Population Center. Integrated Public Use Microdata Series, International: Version 6.1 [Machine-readable database]. Minneapolis: University of Minnesota, 2011). The data has been recorded by the Uganda Bureau of Statistics (sampling day was September 12, 2002). The census was conducted by face-to-face interviews; enumeration unit was the household. Women aged between 12 and 54 years (Table S1) were asked first, how many children they have born, secondly, how many male children and female children they have born. These data provide no information on birth order; it only includes information on the number of children.

For each mother in our sample, we calculated the sex ratio of her born children. In total, 438,640 mothers were included. We did not restrict our analysis to women who already have completed their reproduction, because an increase in the number of male offspring may be related to earlier mortality of mothers compared with those who gave birth to more girls [43]. Therefore, restricting of our analyses for example, to women who did complete reproduction 45 years and older may bias our results towards mothers who had given birth more frequently to girls.

In Figure S1 we show that the age distribution of mothers is skewed to the left - consequently to younger ages.

We calculated the overall sex ratio of all children per mother as follows: Number of males divided by the (number of males+number of females). Ownership of dwelling was encoded as 1 if the dwelling is owned by a household member and as 2 if the dwelling is not owned by a household member. Ownership of dwelling is associated with increased age of mothers (Figure S2) and the number of offspring is related with increased age (Figure S3). To control for potential age effects on SRB we divided our sample into eight age intervals (12 to 16; 17 to 21; 22 to 26; 27 to 31; 32 to 36; 37 to 41; 42 to 46; 47 to 54): Mean SRB values (± SE) are plotted for each interval under the assumption of dwelling owned or not owned by a household member.

### General Linear Mixed Models (LMM)

Two LMM were calculated on basis of a normal error structure with age, educational attainment (categorized in four levels, one = less than primary completed; two = primary completed; three = secondary completed; four = university completed), marital status (encoded one = single or never married; two = married or living in union; three = separated or divorced or spouse is absent; four = widowed) and ownership of dwelling (owned vs. not owned) as fixed factor and ethnicity, respectively, district of Uganda as random factor regressing on SRB. A second model on basis of a normal error structure with age, education, type of marriage (polygamous vs. monogamous), ownership or non-ownership of dwelling as fixed factor, ethnicity and district of Uganda as random factor regressing on SRB as well (see Tables S2, S3, S4, S5, S6).

### Generalized Linear Models (GLM)

For regressing on the number of born children, we calculated a GLM on basis of a quasi-poisson error structure using age, education, type of marriage, ownership and non-ownership of dwelling as explaining variables. In a further model regressing on the number of born children on basis of a quasi-poisson error structure with age, education, marital status (please see above), ownership and non-ownership of dwelling as explaining variables.

For statistical validation of these results we calculated two separate generalized linear models on basis of a binomial error structure [44] of i) age, education, marital status, regressing on the proportion of male and female births and ii) age, education, type of marriage, regressing on the proportion of male and female births. For both models we show parameter estimates, significances and odds ratios in Table S7 and S8.

## Supporting Information

Figure S1 **The age structure of all reproductive active women is shown (at least one is child born).**
(TIF)

Figure S2 **The age distribution of reproductive active women who owned a dwelling is shown.**
(TIF)

Figure S3 **The mean offspring count of reproductive active women in relation to their age is presented (values are mean ± SE).**
(TIF)

Table S1 **Age distribution of reproducing women.**
(DOC)

Table S2 **Distribution of dwelling ownership (only women who did reproduce).**
(DOC)

Table S3 **Educational attainment (only women who did reproduce).**
(DOC)

Table S4 **Marital status (only women who did reproduce).**
(DOC)

Table S5 **Frequency of polygamy (only for women who are married and did reproduce).**
(DOC)

Table S6 **Frequencies and descriptive statistics of offspring count for all women older than 45.**
(DOC)

Table S7 **Age, education, marital status and dwelling ownership, regressing on the proportion of male and female births on basis of a binomial error structure: parameter estimates, standard errors, significances and odds ratios.**
(DOC)

Table S8 **Age, education, type of marriage and dwelling ownership, regressing on the proportion of male and female births on basis of a binomial error structure: parameter estimates, standard errors, significances and odds ratios.**
(DOC)
