# Supplementary figures and images for: Ownership of Dwelling Affects the Sex Ratio at Birth in Uganda
Source: PLoS One. 2012 Dec 17;7(12):e51463. doi: 10.1371/journal.pone.0051463 (PMC3524175; doi:10.1371/journal.pone.0051463)

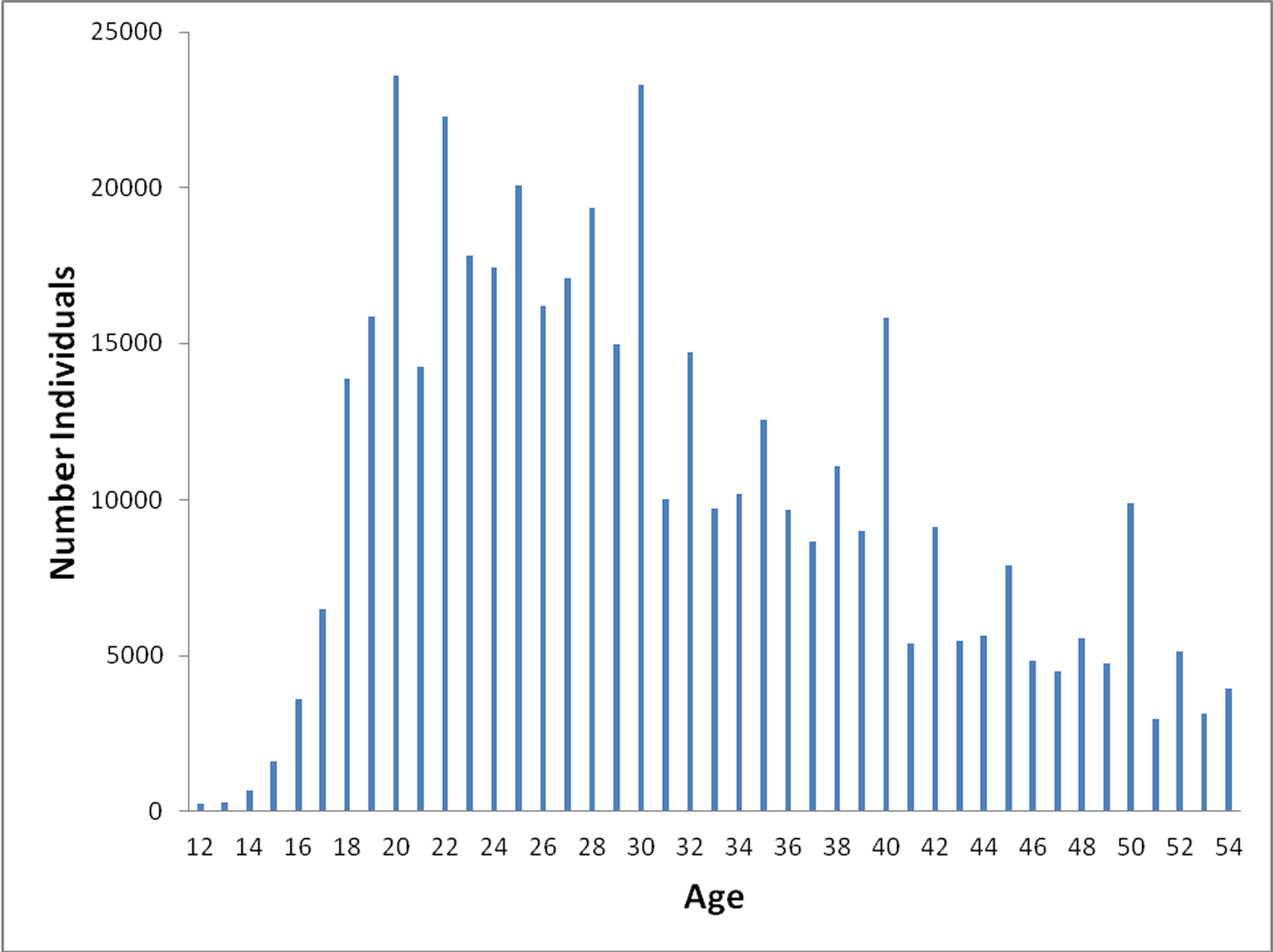

Supplement: Figure S1 — The age structure of all reproductive active women is shown (at least one is child born). (TIF) [file pone.0051463.s001.tif]

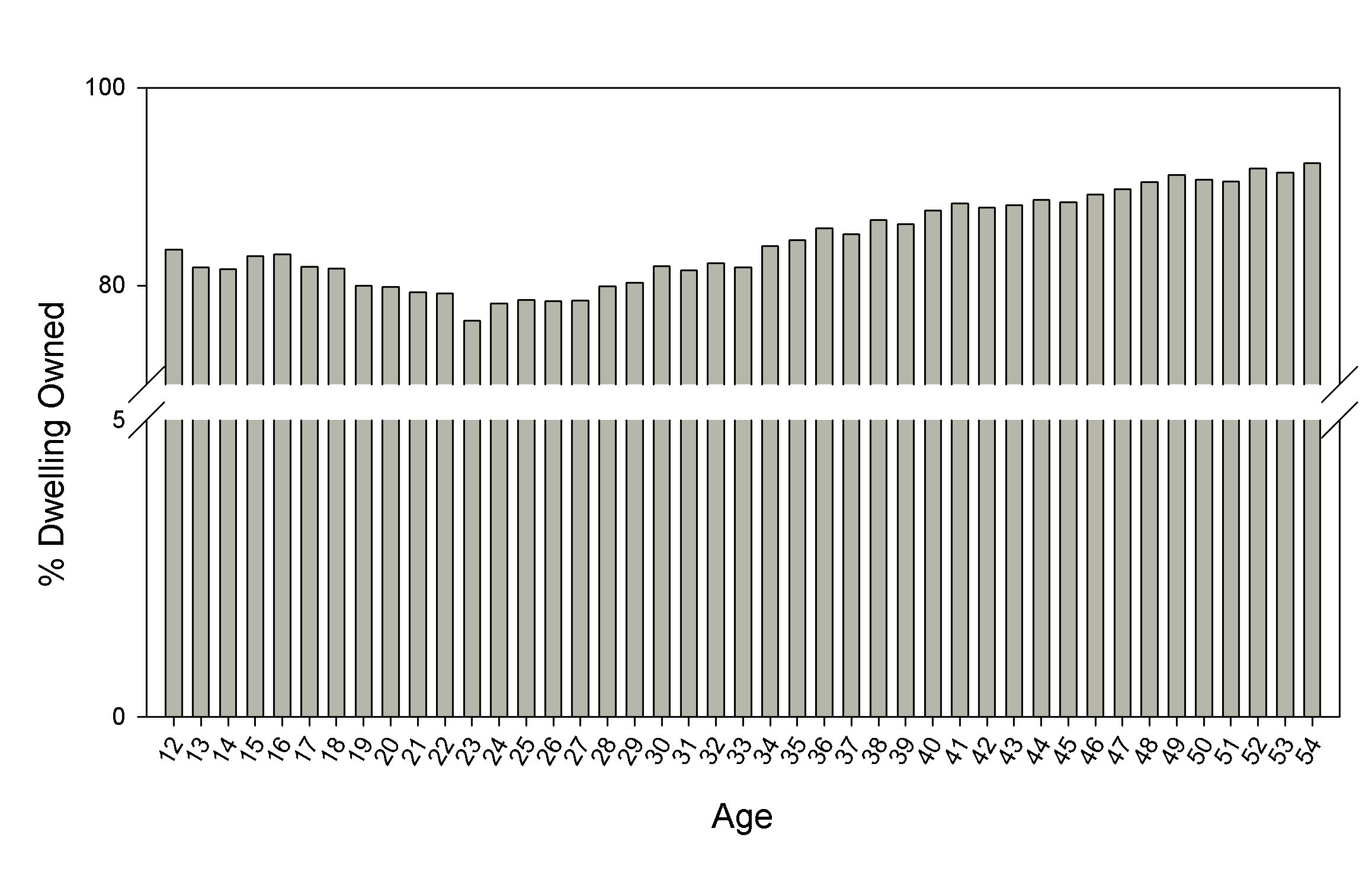

Supplement: Figure S2 — The age distribution of reproductive active women who owned a dwelling is shown. (TIF) [file pone.0051463.s002.tif]
