# Supplementary material for: Ownership of Dwelling Affects the Sex Ratio at Birth in Uganda
Source: PLoS One. 2012 Dec 17;7(12):e51463. doi: 10.1371/journal.pone.0051463 (PMC3524175; doi:10.1371/journal.pone.0051463)
Supplement: Table S1 — Age distribution of reproducing women. (DOC) [file pone.0051463.s004.doc]

|  | | Frequency | Percent | Valid Percent | Cumulative Percent |
| --- | --- | --- | --- | --- | --- |
| Valid | 12 | 229 | .1 | .1 | .1 |
|  | 13 | 264 | .1 | .1 | .1 |
|  | 14 | 664 | .2 | .2 | .3 |
|  | 15 | 1581 | .4 | .4 | .6 |
|  | 16 | 3619 | .8 | .8 | 1.4 |
|  | 17 | 6512 | 1.5 | 1.5 | 2.9 |
|  | 18 | 13857 | 3.2 | 3.2 | 6.1 |
|  | 19 | 15848 | 3.6 | 3.6 | 9.7 |
|  | 20 | 23600 | 5.4 | 5.4 | 15.1 |
|  | 21 | 14252 | 3.3 | 3.3 | 18.3 |
|  | 22 | 22265 | 5.1 | 5.1 | 23.4 |
|  | 23 | 17811 | 4.1 | 4.1 | 27.5 |
|  | 24 | 17426 | 4.0 | 4.0 | 31.5 |
|  | 25 | 20070 | 4.6 | 4.6 | 36.0 |
|  | 26 | 16210 | 3.7 | 3.7 | 39.7 |
|  | 27 | 17108 | 3.9 | 3.9 | 43.6 |
|  | 28 | 19336 | 4.4 | 4.4 | 48.0 |
|  | 29 | 14970 | 3.4 | 3.4 | 51.5 |
|  | 30 | 23289 | 5.3 | 5.3 | 56.8 |
|  | 31 | 10022 | 2.3 | 2.3 | 59.1 |
|  | 32 | 14723 | 3.4 | 3.4 | 62.4 |
|  | 33 | 9730 | 2.2 | 2.2 | 64.6 |
|  | 34 | 10195 | 2.3 | 2.3 | 67.0 |
|  | 35 | 12541 | 2.9 | 2.9 | 69.8 |
|  | 36 | 9666 | 2.2 | 2.2 | 72.0 |
|  | 37 | 8663 | 2.0 | 2.0 | 74.0 |
|  | 38 | 11055 | 2.5 | 2.5 | 76.5 |
|  | 39 | 8998 | 2.1 | 2.1 | 78.6 |
|  | 40 | 15805 | 3.6 | 3.6 | 82.2 |
|  | 41 | 5375 | 1.2 | 1.2 | 83.4 |
|  | 42 | 9119 | 2.1 | 2.1 | 85.5 |
|  | 43 | 5460 | 1.2 | 1.2 | 86.7 |
|  | 44 | 5648 | 1.3 | 1.3 | 88.0 |
|  | 45 | 7897 | 1.8 | 1.8 | 89.8 |
|  | 46 | 4819 | 1.1 | 1.1 | 90.9 |
|  | 47 | 4480 | 1.0 | 1.0 | 91.9 |
|  | 48 | 5543 | 1.3 | 1.3 | 93.2 |
|  | 49 | 4761 | 1.1 | 1.1 | 94.3 |
|  | 50 | 9913 | 2.3 | 2.3 | 96.5 |
|  | 51 | 2958 | .7 | .7 | 97.2 |
|  | 52 | 5115 | 1.2 | 1.2 | 98.4 |
|  | 53 | 3135 | .7 | .7 | 99.1 |
|  | 54 | 3928 | .9 | .9 | 100.0 |
|  | Total | 438460 | 100.0 | 100.0 |  |
