# Supplementary material for: Ownership of Dwelling Affects the Sex Ratio at Birth in Uganda
Source: PLoS One. 2012 Dec 17;7(12):e51463. doi: 10.1371/journal.pone.0051463 (PMC3524175; doi:10.1371/journal.pone.0051463)
Supplement: Table S2 — Distribution of dwelling ownership (only women who did reproduce). (DOC) [file pone.0051463.s005.doc]

|  | | Frequency | Percent | Valid Percent | Cumulative Percent |
| --- | --- | --- | --- | --- | --- |
| Valid | Owned | 359926 | 82.1 | 82.7 | 82.7 |
|  | Not owned | 75456 | 17.2 | 17.3 | 100.0 |
|  | Total | 435382 | 99.3 | 100.0 |  |
