# Supplementary material for: Ownership of Dwelling Affects the Sex Ratio at Birth in Uganda
Source: PLoS One. 2012 Dec 17;7(12):e51463. doi: 10.1371/journal.pone.0051463 (PMC3524175; doi:10.1371/journal.pone.0051463)
Supplement: Table S3 — Educational attainment (only women who did reproduce). (DOC) [file pone.0051463.s006.doc]

|  | | Frequency | Percent | Valid Percent | Cumulative Percent |
| --- | --- | --- | --- | --- | --- |
| Valid | Less than primary completed | 277931 | 63.4 | 63.4 | 63.4 |
|  | Primary completed | 143647 | 32.8 | 32.8 | 96.1 |
|  | Secondary completed | 15096 | 3.4 | 3.4 | 99.6 |
|  | University completed | 1786 | .4 | .4 | 100.0 |
|  | Total | 438460 | 100.0 | 100.0 |  |
