# Supplementary material for: Ownership of Dwelling Affects the Sex Ratio at Birth in Uganda
Source: PLoS One. 2012 Dec 17;7(12):e51463. doi: 10.1371/journal.pone.0051463 (PMC3524175; doi:10.1371/journal.pone.0051463)
Supplement: Table S4 — Marital status (only women who did reproduce). (DOC) [file pone.0051463.s007.doc]

|  | | Frequency | Percent | Valid Percent | Cumulative Percent |
| --- | --- | --- | --- | --- | --- |
| Valid | Single/never married | 42864 | 9.8 | 9.8 | 9.8 |
|  | Married/in union | 334074 | 76.2 | 76.2 | 86.0 |
|  | Separated/divorced/spouse absent | 33992 | 7.8 | 7.8 | 93.7 |
|  | Widowed | 27530 | 6.3 | 6.3 | 100.0 |
|  | Total | 438460 | 100.0 | 100.0 |  |
