# Supplementary material for: Ownership of Dwelling Affects the Sex Ratio at Birth in Uganda
Source: PLoS One. 2012 Dec 17;7(12):e51463. doi: 10.1371/journal.pone.0051463 (PMC3524175; doi:10.1371/journal.pone.0051463)
Supplement: Table S5 — Frequency of polygamy (only for women who are married and did reproduce). (DOC) [file pone.0051463.s008.doc]

|  | | Frequency | Percent | Valid Percent | Cumulative Percent |
| --- | --- | --- | --- | --- | --- |
| Valid | No, in monogamous union | 269859 | 80.8 | 80.8 | 80.8 |
|  | Yes, in polygamous union | 64215 | 19.2 | 19.2 | 100.0 |
|  | Total | 334074 | 100.0 | 100.0 |  |
