# Supplementary material for: Ownership of Dwelling Affects the Sex Ratio at Birth in Uganda
Source: PLoS One. 2012 Dec 17;7(12):e51463. doi: 10.1371/journal.pone.0051463 (PMC3524175; doi:10.1371/journal.pone.0051463)
Supplement: Table S6 — Frequencies and descriptive statistics of offspring count for all women older than 45. (DOC) [file pone.0051463.s009.doc]

|  | | Frequency | Percent | Valid Percent | Cumulative Percent |
| --- | --- | --- | --- | --- | --- |
| Valid | .00 | 2536 | 1.0 | 5.4 | 5.4 |
|  | 1.00 | 1822 | .7 | 3.9 | 9.2 |
|  | 2.00 | 2207 | .9 | 4.7 | 13.9 |
|  | 3.00 | 2387 | 1.0 | 5.1 | 19.0 |
|  | 4.00 | 2948 | 1.2 | 6.2 | 25.2 |
|  | 5.00 | 3367 | 1.4 | 7.1 | 32.4 |
|  | 6.00 | 3865 | 1.6 | 8.2 | 40.5 |
|  | 7.00 | 4330 | 1.8 | 9.2 | 49.7 |
|  | 8.00 | 4954 | 2.0 | 10.5 | 60.2 |
|  | 9.00 | 4646 | 1.9 | 9.8 | 70.1 |
|  | 10.00 | 4811 | 2.0 | 10.2 | 80.3 |
|  | 11.00 | 3289 | 1.3 | 7.0 | 87.2 |
|  | 12.00 | 2621 | 1.1 | 5.6 | 92.8 |
|  | 13.00 | 1518 | .6 | 3.2 | 96.0 |
|  | 14.00 | 803 | .3 | 1.7 | 97.7 |
|  | 15.00 | 500 | .2 | 1.1 | 98.8 |
|  | 16.00 | 295 | .1 | .6 | 99.4 |
|  | 17.00 | 129 | .1 | .3 | 99.7 |
|  | 18.00 | 108 | .0 | .2 | 99.9 |
|  | 19.00 | 52 | .0 | .1 | 100.0 |
|  | Total | 47188 | 19.2 | 100.0 |  |
| Missing | System | 198661 | 80.8 |  |  |
| Total | | 245849 | 100.0 |  |  |

| N | Valid | 47188 |
| --- | --- | --- |
|  | Missing | 198661 |
| Mean | | 7.2271 |
| Std. Error of Mean | | .01764 |
| Median | | 8.0000 |
| Percentiles | 25 | 4.0000 |
|  | 50 | 8.0000 |
|  | 75 | 10.0000 |
