# Supplementary material for: Ownership of Dwelling Affects the Sex Ratio at Birth in Uganda
Source: PLoS One. 2012 Dec 17;7(12):e51463. doi: 10.1371/journal.pone.0051463 (PMC3524175; doi:10.1371/journal.pone.0051463)
Supplement: Table S7 — Age, education, marital status and dwelling ownership, regressing on the proportion of male and female births on basis of a binomial error structure: parameter estimates, standard errors, significances and odds ratios. (DOC) [file pone.0051463.s010.doc]

|  |  |  |  |  | **95%** | **Conf.** |
| --- | --- | --- | --- | --- | --- | --- |
|  | **Estimate** | **SE** | **P** | **Odds ratio** | **Lower limit** | **Upper limit** |
| (Intercept) | -0.025 | 0.008 | 0.002 | 0.975 | -0.0407895852 | -0.0095945920 |
| age | 0.000 | 0.000 | 0.759 | 1.000 | -0.0002618602 | 0.0003588526 |
| primary completed (ref. less than primary completed) | 0.006 | 0.003 | 0.074 | 1.006 | -0.0005721622 | 0.0121984740 |
| secondary completed (ref. less than primary completed) | 0.007 | 0.010 | 0.469 | 1.007 | -0.0118414283 | 0.0257307964 |
| university completed (ref. less than primary completed) | -0.032 | 0.028 | 0.244 | 0.968 | -0.0864389293 | 0.0220036647 |
| married or living in union (ref. single or never married) | 0.024 | 0.007 | 0.001 | 1.024 | 0.0106546597 | 0.0367317147 |
| separated or divorced or spouse is absent (ref. single or never married) | 0.016 | 0.008 | 0.059 | 1.016 | -0.0005765761 | 0.0322084187 |
| widowed (ref. single or never married) | 0.014 | 0.008 | 0.092 | 1.014 | -0.0023063451 | 0.0305110205 |
| dwelling not owned (dwelling owned ref. ) | -0.024 | 0.004 | 0.001 | 0.977 | -0.0322898705 | -0.0152680191 |
| residual deviance: 545185 on 435373 degrees of freedom |  |  |  |  |  |  |
