# Supplementary material for: Ownership of Dwelling Affects the Sex Ratio at Birth in Uganda
Source: PLoS One. 2012 Dec 17;7(12):e51463. doi: 10.1371/journal.pone.0051463 (PMC3524175; doi:10.1371/journal.pone.0051463)
Supplement: Table S8 — Age, education, type of marriage and dwelling ownership, regressing on the proportion of male and female births on basis of a binomial error structure: parameter estimates, standard errors, significances and odds ratios. (DOC) [file pone.0051463.s011.doc]

|  |  |  |  |  | **95%** | **Conf.** |
| --- | --- | --- | --- | --- | --- | --- |
|  | **Estimate** | **SE** | **P** | **Odds ratio** | **Lower limit** | **Upper limit** |
| (Intercept) | 0.004 | 0.007 | 0.539 | 0.975 | -0.0088993772 | 0.0170373913 |
| age | 0.000 | 0.000 | 0.806 | 1.000 | -0.0003915287 | 0.0003042898 |
| primary completed (ref. less than primary completed) | 0.004 | 0.004 | 0.222 | 1.006 | -0.0027213110 | 0.0117128828 |
| secondary completed (ref. less than primary completed) | 0.005 | 0.012 | 0.665 | 1.007 | -0.0175829231 | 0.0275743212 |
| university completed (ref. less than primary completed) | 0.001 | 0.033 | 0.967 | 0.968 | -0.0628066545 | 0.0655185558 |
| polygamous union (ref. monogamous) | -0.010 | 0.004 | 0.008 | 1.024 | -0.0179216593 | -0.0026388199 |
| dwelling not owned (dwelling owned ref. ) | -0.023 | 0.005 | 0.001 | 0.977 | -0.0326413209 | -0.0125601847 |
| residual deviance: 412471 on 332152 degrees of freedom |  |  |  |  |  |  |
